# Neuronal Replacement in Stem Cell Therapy for Stroke: Filling the Gap

**DOI:** 10.3389/fcell.2021.662636

**Published:** 2021-04-06

**Authors:** Sara Palma-Tortosa, Berta Coll-San Martin, Zaal Kokaia, Daniel Tornero

**Affiliations:** ^1^Laboratory of Stem Cells and Restorative Neurology, Lund Stem Cell Center, Lund University, Lund, Sweden; ^2^Department of Biomedical Sciences, Institute of Neuroscience and Production and Validation Center of Advanced Therapies (Creatio), University of Barcelona, Barcelona, Spain; ^3^August Pi I Sunyer Biomedical Research Institute (IDIBAPS), Barcelona, Spain

**Keywords:** stem cell therapy, stroke, cell replacement, functional integration, neural stem cells

## Abstract

Stem cell therapy using human skin-derived neural precursors holds much promise for the treatment of stroke patients. Two main mechanisms have been proposed to give rise to the improved recovery in animal models of stroke after transplantation of these cells. First, the so called by-stander effect, which could modulate the environment during early phases after brain tissue damage, resulting in moderate improvements in the outcome of the insult. Second, the neuronal replacement and functional integration of grafted cells into the impaired brain circuitry, which will result in optimum long-term structural and functional repair. Recently developed sophisticated research tools like optogenetic control of neuronal activity and rabies virus monosynaptic tracing, among others, have made it possible to provide solid evidence about the functional integration of grafted cells and its contribution to improved recovery in animal models of brain damage. Moreover, previous clinical trials in patients with Parkinson’s Disease represent a proof of principle that stem cell-based neuronal replacement could work in humans. Our studies with *in vivo* and *ex vivo* transplantation of human skin-derived cells neurons in animal model of stroke and organotypic cultures of adult human cortex, respectively, also support the hypothesis that human somatic cells reprogrammed into neurons can get integrated in the human lesioned neuronal circuitry. In the present short review, we summarized our data and recent studies from other groups supporting the above hypothesis and opening new avenues for development of the future clinical applications.

## Introduction

Ischemic stroke leads to neuronal loss due to shortage of glucose and oxygen supply to an area of the brain, being one of the leading causes of death and adult disability worldwide. The mechanical thrombectomy and pharmacological thrombolysis are the only approved treatments, which are focused on the elimination of the clogging agents in the acute phase after the insult (up to 6 h), making only this fraction of patients eligible for treatment. Unfortunately, nowadays there are virtually no treatments to support efficient recovery of impaired sensory, motor, and cognitive deficits in stroke-surviving patients and more than half of them remain disabled to various degree. Therefore, there is a high demand on new strategies that will support the spontaneously occurring regeneration of damaged neuronal tissue and lead to a more efficient long-term recovery.

In this regard, transplantation of specific types of neuronal precursors/progenitors is an emerging and promising therapy for stroke patients, which has been pioneered in the treatment of Parkinson Disease (PD) using dopaminergic neurons from aborted human fetuses (Lindvall et al., 1990; Kordower et al., 1998). Many studies using animal models mimicking different neurological conditions with brain damage have shown that stem cell-based treatment might improve recovery through two types of action mode. First mode, the so-called bystander effect, is proposed to be caused by release of different factors leading to immunomodulation, reduction of brain-blood-barrier damage (Eckert et al., 2015), stimulation of angiogenesis, endogenous neurogenesis, and neuronal plasticity (Chang et al., 2013; Mine et al., 2013). The second mode is based on cell replacement and justified by recent publications demonstrating the ability of grafted pluripotent stem cells (PSCs) to morphologically differentiate into different types of neurons, establish synaptic connections with the host circuitry and get integrated in damaged neuronal network (Grade and Gotz, 2017).

Bystander effect, the first action mode, could only have moderate effect on the improvement of spontaneous regeneration through modulation of tissue environment. This could be even less effective in the brain of elderly patients, which represent the majority of the ones suffering stroke, due to a decreased cellular plasticity and self-repair capacity. On contrary, supporting brain with young neurons for replacement of dead or damaged ones could be more efficient mode of action and might lead to better, long-term and sustainable recovery of impaired functions.

At the end of the 90’s, the capacity to isolate and culture human embryonic stem cells (ESCs) (Thomson et al., 1998), opened the possibility to generate more easily and robustly specific neuronal subtypes for regenerative medicine, avoiding several problems related to the use of human fetal tissue. One decade later, thanks to the advent of cell reprogramming technology, human adult somatic cells (i.e., skin cells) can be converted into induced PSCs (iPSCs) (Yamanaka, 2007), allowing generation of patient-specific neurons. The use of human iPSCs for cell therapy avoids need for long immunosuppressive treatments, risk of graft rejection and ethical concerns related to the use of human embryos. In this regard, it has been recently shown that dopaminergic progenitors derived from clinical-grade human ESC or iPSC lines are safe and effective for cell-based therapy in PD (Doi et al., 2020; Kim et al., 2020). Most importantly, human iPSC transplantation into the putamen of a PD patient suggested graft survival as well as improvement of PD symptoms at 18–24 months after surgery (Schweitzer et al., 2020). These encouraging results motivate the use of cell replacement strategy not only for PD but also for other neurological disorders such as stroke. In contrast to PD, stroke affects different neuronal cell types depending on the size and the location of the injury, which has required the development of very specific differentiation protocols to generate the adequate cell population for transplantation (Alia et al., 2019). Besides this, new technological tools have allowed the confirmation of neuronal replacement after transplantation of different kinds of PSC-derived neural precursors into the damaged brain as is summarized in the present review. These advances bring stem cell therapy closer to a clinical application for stroke patients.

## By the Hands of Technology

**FIGURE 1:**
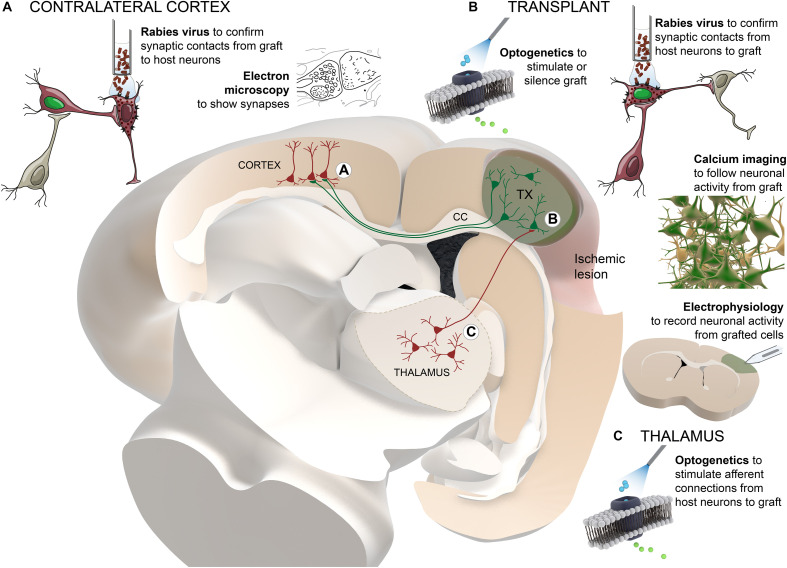
Schematic summary of the technological approaches used to study the functional connectivity between grafted and host neurons. Transplanted cells (colored in green) are located adjacent to the cortical ischemic lesion. For clarity and as an example, thalamo-cortical afferent projections to the graft and transcallosal projections from graft to host cortical neurons in the contralateral hemisphere are presented. Different techniques are distributed in the areas of the brain where they are applied: **(A)** contralateral cortex, **(B)** transplant, **(C)** thalamus. TX, transplant; CC, corpus callosum.

Solid evidence demonstrating that graft-derived neurons can functionally integrate in host brain circuitry has been closely related to the development of new technological tools. Monosynaptic tracing of neurons using rabies virus, control and monitoring of neuronal activity by optogenetic technology and recording of intracellular calcium levels, respectively, together with more “traditional” techniques such as electron microscopy or electrophysiological recordings, allowed thorough study of functional connectivity between grafted and host neurons (Figure 1).

Rabies virus has the ability to spread transynaptically between neurons. The connected neurons can be genetically targeted using tracing vectors with neuronal promoters and receptors for specific viral envelopes (Wickersham et al., 2007). Pseudotyped rabies virus carrying fluorescent dyes can be used for identifying either cells with presynaptic connection to grafted cells (Grealish et al., 2015; Tornero et al., 2017) or brain areas where host neurons have received synaptic contacts from the grafted neurons (Palma-Tortosa et al., 2020). New improved methods for brain tissue clearing, such as iDISCO or CUBIC, have given the possibility to perform fluorescence imaging in 3D stained tissue, preserving anatomical structures in whole brain or a portion of it, allowing the detection of graft projections and monosynaptic input maps without the need for less accurate sectioning and further reconstruction of the tissue (Doerr et al., 2017). Unfortunately, the resolution of these imaging techniques does not allow visualization of projections or connections at the level of individual cell, but they have shown great potential for a general overview of the distribution of cells and their axonal projections.

Once the location of host neurons innervating grafted cells is detected, functionality of their connections can be addressed using traditional extracellular electrophysiological recordings in alive anesthetized animals in response to afferent stimulation (Tornero et al., 2017). As an alternative, optogenetic tools have been used to modify electrophysiological properties of specific set of neurons that express opsin proteins sensitive to light of particular wavelength (Cheng et al., 2014). Similarly, the so-called DREADDs (from designer receptors exclusively activated by designer drugs), uses inert chemical ligands to activate or inhibit neuronal activity (Armbruster et al., 2007). These last two strategies can be combined with *in vivo* electrophysiology as well as with patch-clamp in acute slices to confirm functional synaptic connectivity between host neurons and grafted cells. Moreover, silencing of grafted cells using optogenetic or DREADD tools allowed to assess the contribution of its neuronal activity to functional recovery (Palma-Tortosa et al., 2020), while the stimulation of graft-derived neurons using a combination of optogenetics and DREADDs (optochemogenetic) can be used to improve its functional integration into the host circuitry (Yu et al., 2019).

On the other hand, calcium imaging using genetically encoded calcium indicators (GECIs) allows quantitative monitoring of neuronal activity in alive animals while exposing them to sensory stimulus (Falkner et al., 2016; Linaro et al., 2019). Together with two-photon microscopy, GECIs enable single cell and high temporal resolution in a three-dimensional framework. Moreover, this approach allows evaluation at multiple time-points using cranial window in the skull of the animals, making it possible to track dynamics of functional integration of grafted neurons. This strategy is limited to monitoring superficial cortical layers, even though technology is advancing rapidly to achieve devices that allow the visualization of deeper areas of the brain.

In the same line, bioluminescence reporters are also a validated method for non-invasive monitoring of grafted cells (Vogel et al., 2019). Despite lower spatial resolution of *in vivo* bioluminescence imaging as compared with calcium imaging, the use of cell-type specific promoters allows the study of cell therapy in animal models. Although this strategy does not provide information about neuronal functional integration, it has shown much potential for the study of the maturation of the different cell types generated by the transplant.

The development of all these techniques has boosted the studies on neuronal replacement after transplantation of PSC-derived cells into the damaged brain, allowing to obtain qualitatively new data for the assessment of this approach in future clinical settings.

## Cell Replacement

Several decades ago, human fetal grafts, enriched with dopaminergic progenitors and transplanted in the striatum of PD patients showed good survival and capacity to improve motor function (Lindvall et al., 1990). The visualization of the graft in post-mortem samples two decades later showed a remarkable level and specificity of circuit integration in the host brain (Li et al., 2016). This was the first solid evidence that functional restoration of damaged neuronal circuitry might be possible using human PSC transplantation. From that moment, the transplantation of PSCs as therapy for other brain disorders, such as ischemic stroke, has progressed from demonstrating high level of specificity when neurons integrate into the damaged circuits, to the confirmation of grafted-cell contribution to functional recovery (studies summarized in Table 1).

**TABLE 1:** Summary of the studies exploring cell replacement occurrence in stem cell therapy for brain damage (organized in chronological order).

References	Cell source	Damage model	Specie	Injection site	Description
Michelsen et al. (2015)	Mouse ESC	Cortical ablation with ibotenic acid	Mouse	Visual cortex	Appropriate cortical area identity of grafted neurons is essential for correct reconstruction of adult damaged cortical circuitry
Falkner et al. (2016)	Mouse Fetal	Cortical ablation with chlorine e6	Mouse	Visual cortex	Neocortical grafted cells integrate structurally and functionally into the adult cortical circuitry
Doerr et al. (2017)	Human ESC	No lesion	Mouse	Hippocampus and Striatum	Innervation network developed by grafted cells is similar to the one generated by endogenous neurons, being determinant the area where the cells are transplanted
Tornero et al. (2017)	Human iPSC	Cortical ischemic stroke	Rat	Sensorimotor cortex	Grafted neurons integrate in stroke-injured brain and receive functional afferent inputs from host neurons that are activated by sensory stimuli
Somaa et al. (2017)	Human ESC	Focal Ischemia with endothelin-1	Rat	Sensorimotor cortex	Hydrogels fabricated with peptides for laminin-derived epitope improve differentiation and enhance synaptic connectivity of human ESC-derived cortical neurons grafted after stroke
Green et al. (2018)	Human ESC	Cortical ischemic stroke	Mouse	Sensorimotor cortex	Grafted neurons stabilize stroke-damaged functional neuronal networks through paracrine effects
Terrigno et al. (2018)	Mouse ESC	No lesion/Ischemic lesion	Mouse	Cortex and Hippocampus	Identity of grafted neuronal precursors determine its connectivity and integration after transplantation in cortex or striatum
Espuny-Camacho et al. (2018)	Mouse ESC	Cortical ablation with ibotenic acid	Mouse	Visual cortex	Grafted neurons with visual identity display similar functional and morphological features from the host neurons and establish a similar projection pattern
Nisbet et al. (2018)	Human ESC	Focal Ischemia with endothelin-1	Rat	Sensorimotor cortex	Peptide-based hydrogels loaded with BDNF increase long-term survival and vascularization of grafted ESC-derived cortical neurons while reducing secondary degeneration
Vogel et al. (2019)	Human iPSC	No lesion	Mouse	Cortex	*In vivo* luminescence imaging of grafted cells is an effective tool to monitor cell differentiation and to detect its spontaneous differentiation into astrocytes and mature neurons
Yu et al. (2019)	Mouse iPSC	Cortical ischemic stroke	Mouse	Sensorimotor cortex	Optochemogenetic stimulation of grafted cells improve rescue of neural network lost connectivity and function after stroke
Linaro et al. (2019)	Human ESC	No lesion	Mouse	Lateral ventricles	Graft-derived cortical neurons integrate in host neuronal network and combine intrinsic human development with host-like activity pattern
Palma-Tortosa et al. (2020)	Human iPSC	Cortical ischemic stroke	Rat	Sensorimotor cortex	Graft-derived cortical neurons send transcallosal projections to the contralateral hemisphere and generate functional synapses with host neurons contributing to behavioral improvements
Andreoli et al. (2020)	Rat Fetal	Cortical ablation with DT system	Rat	Sensorimotor cortex	Graft-derived neurons form vascularized clusters that integrate into host circuitry and survive long-term, leading to functional recovery
Xiong et al. (2021)	Human ESC	Parkinson Disease	Mouse	Substantia nigra/Striatum	Graft-derived neurons resemble host ones and its projection pattern depends on intrinsic cell properties. These cells repair nigro-striatal lesioned circuit restoring circuit functionality

*ESC, embryonic stem cell; iPSC, induced pluripotent stem cell; DT, Diphtheria toxin; BDNF, brain-derived neurotrophic factor.*

PSC-derived progenitors transplanted in different animal models of brain damage have proven to differentiate into specific neuronal subtypes and integrate into the host brain circuitry. Moreover, a strong association between graft integration and functional restoration, evidencing the importance of areal-identity match for successful repair, has been shown by several groups (Espuny-Camacho et al., 2018; Terrigno et al., 2018; Xiong et al., 2021). Given this, substantial progress has been made in optimizing protocols for the generation of scalable cell populations produced under standardized and quality-controlled conditions for future therapeutic use (Steinbeck and Studer, 2015). Most commonly used protocols for neuralization of human ESCs or iPSCs combine small-molecule inhibitors of bone morphogenic protein (BMP) and TGFβ/activin/nodal signals (Morizane et al., 2011; Qi et al., 2017). This step may or not imply the formation of embryoid bodies, and resulting cells acquire rosette-like morphology (Falk et al., 2012) similar to neuroepithelial cells of the brain. Then, specification of neuronal cell subtypes requires a timed addition of other pattering factors that activate or inhibit master developmental pathways (Petros et al., 2011).

Both, ESC- or iPSC-derived cortical neurons transplanted into the peri-infarct region of damaged visual cortex have been demonstrated to develop a pattern of connectivity similar to endogenous neurons from this area of the brain (Michelsen et al., 2015; Falkner et al., 2016; Espuny-Camacho et al., 2018; Green et al., 2018). Functional studies performed in the same scenario using calcium imaging revealed that grafted neurons were able to respond to specific visual stimuli (Falkner et al., 2016). Supporting the formation of functional afferent connections, iPSC and ESC-derived grafted neurons transplanted into the somatosensory injured cortex after ischemic stroke or specific ablation of layer II and III, respectively, were able to respond to optogenetic activation of thalamic afferent axons as well as to physiological sensory stimuli (Tornero et al., 2017; Andreoli et al., 2020).

Similar results have been found when the transplantation was performed in intact animals. Interestingly, graft-derived neurons with a specific identity (i.e., hippocampal, cortical, or striatal) project to the same target areas than host neurons with the same identity (Doerr et al., 2017; Terrigno et al., 2018). Most importantly, grafted cells display similar activity and tuned responses to sensory stimuli resembling host neurons (Linaro et al., 2019). Also, in animal models affecting other areas of the central nervous system, such as spinal cord injury, it has been shown that transplantation of human iPSC-derived neuronal progenitors gives rise to an improved recovery with functional integration of grafted cells (Nori et al., 2011; Lu et al., 2012).

Convincing demonstration of neuronal replacement should also include evidences of the establishment of functional efferent connections from grafted cells to the neurons of appropriate host brain structures. In this regard, rabies virus monosynaptic tracing has shown that iPSC-derived progenitors transplanted into the somatosensory damaged cortex formed functional efferent synaptic inputs with host neurons located in the contralateral somatosensory cortex (Palma-Tortosa et al., 2020). Importantly, grafted cells traced with rabies virus were positive for specific markers of transcallosal projection neurons located in the healthy cortex. In addition, studies with electron microscopy showed that graft-derived axons exhibited ultrastructural features similar to those of host axons as well as different degrees of myelination by host oligodendrocytes. Regarding the contribution of transplanted iPSC-derived progenitors in the animal motor performance, optogenetic inhibition of the graft-derived neurons at 6 months after transplantation reduced the mobility of both paws in the animals, demonstrating that the graft became part of the host circuitry participating in the physiological motor behavior (Palma-Tortosa et al., 2020).

With the aim of maximizing functional recovery and integration of grafted cells, it has been demonstrated that specific activation of graft-derived neurons by using opto- or chemogenetics induces a considerable improvement including increased neurite outgrowth and synaptic plasticity, upregulation of synaptic proteins, promotion of neuronal differentiation and axonal remyelination (Yu et al., 2019; Andreoli et al., 2020). In the same line, the use of hydrogels functionalized for a laminin-derived epitope mimicking brain extracellular matrix, improves neuronal differentiation and functional electrophysiological properties of human ESC-derived cortical neurons transplanted in an animal stroke model (Somaa et al., 2017). Moreover, loading those hydrogels with brain-derived neurotrophic factor (BDNF), grafted ESC-derived neurons showed enhanced long-term survival and vascularization as well as reduced secondary degeneration of host cortex in the same model (Nisbet et al., 2018).

However, even giving the importance of these studies, they have all been conducted using xenotransplantation of human cells in animal models of neurological disorders. Addressing this issue, recent publication from our group has demonstrated that human iPSC-derived cortical progenitors transplanted into human adult cortical tissue, not only survive and give rise to mature cortical neurons, but also exhibit electrophysiological and ultrastructural properties of functional neurons and establish afferent and efferent synaptic connections with the host human cortical neurons, as evidenced by both, rabies virus monosynaptic tracing and electron microscopy (Gronning Hansen et al., 2020). This is the first evidence that human PSCs can integrate into adult human neural network and supports the potential clinical use of PSCs to restore neuronal damaged network in patient with brain disorders.

## Concluding Remarks and Future Perspectives

Since the potential use of stem cell therapy in stroke patients was suggested for improved recovery of functional deficits, many efforts have been made for better understanding of the mechanisms behind its beneficial effects (Grade and Gotz, 2017; Kokaia et al., 2017). During the last 5 years, several studies with transplantation of stem cell-derived neural progenitors in animal models of different kinds of brain damage have shown the generation of specific synaptic contacts from host to graft and vice versa that are able to send relevant information. These studies highlight the importance of cell specification to generate the very exact subtype of neuronal identity for reconstruction of each brain area. Another limitation for the clinical application of cell-based treatments is that extensive *in vitro* expansion of cellular products is associated with increased genomic and epigenomic instability, urging for standardized cell culture settings to minimize genomic alterations.

In this respect, our group has recently demonstrated using rat stroke model and long-term neuroepithelial-like cells from human origin that neuronal activity from grafted cells participate in the maintenance of normal motor function, allegedly contributing to the improved recovery of the animals (Palma-Tortosa et al., 2020). Moreover, our recent study using organotypic cultures of adult human cortex showed that similar integration occurs also in a human-to-human transplantation setting (Gronning Hansen et al., 2020).

This progress brings stem cell therapy closer to its clinical application for stroke patients. However, further experimental research will be needed to develop protocols for the generation of the optimum cell type with the aim of inducing maximum recovery. Some work has been already done at this level in the context of the generation of dopaminergic progenitors for the treatment of PD (Doi et al., 2020; Kim et al., 2020). Due to the high cost of production of patient-specific cells for regenerative therapies, the future strategies, most likely will be based in human leukocyte antigen (HLA)-typed iPSCs or engineered “universal” ESC lines, to avoid need for immunosuppression (Doi et al., 2020; Piao et al., 2021). Currently, the idea of generating cell banks from which therapeutic products can be derived and matched immunologically to patients has been already proposed (Sullivan et al., 2020; Takahashi, 2020).

To move toward future clinical trials, it would be critical to develop the proper strategy for the selection of stroke patients based on the location and the size of the ischemic lesion, since this can determine the efficacy of the treatment. It should be also pointed out that stroke patients are in most of the cases elderly people, which might influence the ability of the transplanted cells to exert recovery-promoting effects. One should be aware, that data obtained using animal models may not necessarily translate into meaningful effects in a clinical setting. Baring this in mind, now we need to explore in parallel with more investigative basic research in order to maximize the efficacy of the therapy. Stem cell therapy has approached a very exciting stage, and clinical translation should consider the critical scientific, regulatory and ethical issues, in a collaborative way between basic scientist and clinicians.

## Author Contributions

SP-T, BC-S, ZK, and DT wrote the manuscript. All authors contributed to the article and approved the submitted version.

## Conflict of Interest

The authors declare that the research was conducted in the absence of any commercial or financial relationships that could be construed as a potential conflict of interest.

## References

[B1] Alia C. Terrigno M. Busti I. Cremisi F. Caleo M. (2019). Pluripotent stem cells for brain repair: protocols and preclinical applications in cortical and hippocampal pathologies. *Front. Neurosci.* 13:684. 10.3389/fnins.2019.00684 31447623 PMC6691396

[B2] Andreoli E. Petrenko V. Constanthin P. E. Contestabile A. Bocchi R. Egervari K. (2020). Transplanted embryonic neurons improve functional recovery by increasing activity in injured cortical circuits. *Cereb. Cortex* 30 4708–4725. 10.1093/cercor/bhaa075 32266929

[B3] Armbruster B. N. Li X. Pausch M. H. Herlitze S. Roth B. L. (2007). Evolving the lock to fit the key to create a family of G protein-coupled receptors potently activated by an inert ligand. *Proc. Natl. Acad. Sci. U.S.A.* 104 5163–5168. 10.1073/pnas.0700293104 17360345 PMC1829280

[B4] Chang D. J. Lee N. Park I. H. Choi C. Jeon I. Kwon J. (2013). Therapeutic potential of human induced pluripotent stem cells in experimental stroke. *Cell Transplant.* 22 1427–1440. 23044029 10.3727/096368912X657314

[B5] Cheng M. Y. Wang E. H. Woodson W. J. Wang S. Sun G. Lee A. G. (2014). Optogenetic neuronal stimulation promotes functional recovery after stroke. *Proc. Natl. Acad. Sci. U.S.A.* 111 12913–12918. 10.1073/pnas.1404109111 25136109 PMC4156770

[B6] Doerr J. Schwarz M. K. Wiedermann D. Leinhaas A. Jakobs A. Schloen F. (2017). Whole-brain 3D mapping of human neural transplant innervation. *Nat. Commun.* 8:14162. 10.1038/ncomms14162 PMC5253698 28102196

[B7] Doi D. Magotani H. Kikuchi T. Ikeda M. Hiramatsu S. Yoshida K. (2020). Pre-clinical study of induced pluripotent stem cell-derived dopaminergic progenitor cells for Parkinson’s disease. *Nat. Commun.* 11:3369. 10.1038/s41467-020-17165-w PMC7338530 32632153

[B8] Eckert A. Huang L. Gonzalez R. Kim H. S. Hamblin M. H. Lee J. P. (2015). Bystander effect fuels human induced pluripotent stem cell-derived neural stem cells to quickly attenuate early stage neurological deficits after stroke. *Stem Cells Transl. Med.* 4 841–851. 10.5966/sctm.2014-0184 26025980 PMC4479618

[B9] Espuny-Camacho I. Michelsen K. A. Linaro D. Bilheu A. Acosta-Verdugo S. Herpoel A. (2018). Human pluripotent stem-cell-derived cortical neurons integrate functionally into the lesioned adult murine visual cortex in an area-specific way. *Cell Rep.* 23 2732–2743. 10.1016/j.celrep.2018.04.094 29847802 PMC5990494

[B10] Falk A. Koch P. Kesavan J. Takashima Y. Ladewig J. Alexander M. (2012). Capture of neuroepithelial-like stem cells from pluripotent stem cells provides a versatile system for in vitro production of human neurons. *PLoS One* 7:e29597. 10.1371/journal.pone.0029597 22272239 PMC3260177

[B11] Falkner S. Grade S. Dimou L. Conzelmann K. K. Bonhoeffer T. Gotz M. (2016). Transplanted embryonic neurons integrate into adult neocortical circuits. *Nature* 539 248–253. 10.1038/nature20113 27783592

[B12] Grade S. Gotz M. (2017). Neuronal replacement therapy: previous achievements and challenges ahead. *NPJ Regen. Med.* 2:29. 10.1038/s41536-017-0033-0 PMC5677983 29302363

[B13] Grealish S. Heuer A. Cardoso T. Kirkeby A. Jonsson M. Johansson J. (2015). Monosynaptic tracing using modified rabies virus reveals early and extensive circuit integration of human embryonic stem cell-derived neurons. *Stem Cell Reports* 4 975–983. 10.1016/j.stemcr.2015.04.011 26004633 PMC4471831

[B14] Green C. Minassian A. Vogel S. Diedenhofen M. Beyrau A. Wiedermann D. (2018). Sensorimotor functional and structural networks after intracerebral stem cell grafts in the ischemic mouse brain. *J. Neurosci.* 38 1648–1661. 10.1523/jneurosci.2715-17.2018 29321138 PMC6705873

[B15] Gronning Hansen M. Laterza C. Palma-Tortosa S. Kvist G. Monni E. Tsupykov O. (2020). Grafted human pluripotent stem cell-derived cortical neurons integrate into adult human cortical neural circuitry. *Stem Cells Transl. Med.* 9 1365–1377. 32602201 10.1002/sctm.20-0134 PMC7581452

[B16] Kim T. W. Koo S. Y. Studer L. (2020). Pluripotent stem cell therapies for parkinson disease: present challenges and future opportunities. *Front. Cell Dev. Biol.* 8:729. 10.3389/fcell.2020.00729 32903681 PMC7438741

[B17] Kokaia Z. Tornero D. Lindvall O. (2017). Transplantation of reprogrammed neurons for improved recovery after stroke. *Prog. Brain Res.* 231 245–263. 10.1016/bs.pbr.2016.11.013 28554399

[B18] Kordower J. H. Freeman T. B. Chen E. Y. Mufson E. J. Sanberg P. R. Hauser R. A. (1998). Fetal nigral grafts survive and mediate clinical benefit in a patient with Parkinson’s disease. *Mov. Disord.* 13 383–393. 10.1002/mds.870130303 9613726

[B19] Li W. Englund E. Widner H. Mattsson B. van Westen D. Latt J. (2016). Extensive graft-derived dopaminergic innervation is maintained 24 years after transplantation in the degenerating parkinsonian brain. *Proc. Natl. Acad. Sci. U.S.A.* 113 6544–6549. 10.1073/pnas.1605245113 27140603 PMC4988567

[B20] Linaro D. Vermaercke B. Iwata R. Ramaswamy A. Libe-Philippot B. Boubakar L. (2019). Xenotransplanted human cortical neurons reveal species-specific development and functional integration into mouse visual circuits. *Neuron* 104 972–986e6. 31761708 10.1016/j.neuron.2019.10.002 PMC6899440

[B21] Lindvall O. Brundin P. Widner H. Rehncrona S. Gustavii B. Frackowiak R. (1990). Grafts of fetal dopamine neurons survive and improve motor function in Parkinson’s disease. *Science* 247 574–577. 10.1126/science.2105529 2105529

[B22] Lu P. Wang Y. Graham L. McHale K. Gao M. Wu D. (2012). Long-distance growth and connectivity of neural stem cells after severe spinal cord injury. *Cell* 150 1264–1273. 10.1016/j.cell.2012.08.020 22980985 PMC3445432

[B23] Michelsen K. A. Acosta-Verdugo S. Benoit-Marand M. Espuny-Camacho I. Gaspard N. Saha B. (2015). Area-specific reestablishment of damaged circuits in the adult cerebral cortex by cortical neurons derived from mouse embryonic stem cells. *Neuron* 85 982–997. 10.1016/j.neuron.2015.02.001 25741724

[B24] Mine Y. Tatarishvili J. Oki K. Monni E. Kokaia Z. Lindvall O. (2013). Grafted human neural stem cells enhance several steps of endogenous neurogenesis and improve behavioral recovery after middle cerebral artery occlusion in rats. *Neurobiol. Dis.* 52 191–203. 10.1016/j.nbd.2012.12.006 23276704

[B25] Morizane A. Doi D. Kikuchi T. Nishimura K. Takahashi J. (2011). Small-molecule inhibitors of bone morphogenic protein and activin/nodal signals promote highly efficient neural induction from human pluripotent stem cells. *J. Neurosci. Res.* 89 117–126. 10.1002/jnr.22547 21162120

[B26] Nisbet D. R. Wang T. Y. Bruggeman K. F. Niclis J. C. Somaa F. A. Penna V. (2018). Shear containment of BDNF within molecular hydrogels promotes human stem cell engraftment and postinfarction remodeling in stroke. *Adv. Biosys.* 2:1800113. 10.1002/adbi.201800113

[B27] Nori S. Okada Y. Yasuda A. Tsuji O. Takahashi Y. Kobayashi Y. (2011). Grafted human-induced pluripotent stem-cell-derived neurospheres promote motor functional recovery after spinal cord injury in mice. *Proc. Natl. Acad. Sci. U.S.A.* 108 16825–16830. 10.1073/pnas.1108077108 21949375 PMC3189018

[B28] Palma-Tortosa S. Tornero D. Gronning Hansen M. Monni E. Hajy M. Kartsivadze S. (2020). Activity in grafted human iPS cell-derived cortical neurons integrated in stroke-injured rat brain regulates motor behavior. *Proc. Natl. Acad. Sci. U.S.A.* 117 9094–9100. 10.1073/pnas.2000690117 32253308 PMC7183146

[B29] Petros T. J. Tyson J. A. Anderson S. A. (2011). Pluripotent stem cells for the study of CNS development. *Front. Mol. Neurosci.* 4:30. 10.3389/fnmol.2011.00030 22016722 PMC3191505

[B30] Piao J. Zabierowski S. Dubose B. N. Hill E. J. Navare M. Claros N. (2021). Preclinical efficacy and safety of a human embryonic stem cell-derived midbrain dopamine progenitor product, MSK-DA01. *Cell Stem Cell* 28 217–229.e7. 33545080 10.1016/j.stem.2021.01.004 PMC7903922

[B31] Qi Y. Zhang X. J. Renier N. Wu Z. Atkin T. Sun Z. (2017). Combined small-molecule inhibition accelerates the derivation of functional cortical neurons from human pluripotent stem cells. *Nat. Biotechnol.* 35 154–163. 10.1038/nbt.3777 28112759 PMC5516899

[B32] Schweitzer J. S. Song B. Herrington T. M. Park T. Y. Lee N. Ko S. (2020). Personalized iPSC-derived dopamine progenitor cells for parkinson’s disease. *N. Engl. J. Med.* 382 1926–1932. 32402162 10.1056/NEJMoa1915872 PMC7288982

[B33] Somaa F. A. Wang T. Y. Niclis J. C. Bruggeman K. F. Kauhausen J. A. Guo H. (2017). Peptide-based scaffolds support human cortical progenitor graft integration to reduce atrophy and promote functional repair in a model of stroke. *Cell Rep.* 20 1964–1977. 10.1016/j.celrep.2017.07.069 28834757

[B34] Steinbeck J. A. Studer L. (2015). Moving stem cells to the clinic: potential and limitations for brain repair. *Neuron* 86 187–206. 10.1016/j.neuron.2015.03.002 25856494 PMC4443446

[B35] Sullivan S. Fairchild P. J. Marsh S. G. E. Muller C. R. Turner M. L. Song J. (2020). Haplobanking induced pluripotent stem cells for clinical use. *Stem Cell Res.* 49:102035. 10.1016/j.scr.2020.102035 33221677

[B36] Takahashi J. (2020). iPS cell-based therapy for parkinson’s disease: a kyoto trial. *Regen Ther* 13 18–22. 10.1016/j.reth.2020.06.002 33490319 PMC7794047

[B37] Terrigno M. Busti I. Alia C. Pietrasanta M. Arisi I. D’Onofrio M. (2018). Neurons generated by mouse ESCs with hippocampal or cortical identity display distinct projection patterns when Co-transplanted in the adult brain. *Stem Cell Rep.* 10 1016–1029. 10.1016/j.stemcr.2018.01.010 29456186 PMC5918192

[B38] Thomson J. A. Itskovitz-Eldor J. Shapiro S. S. Waknitz M. A. Swiergiel J. J. Marshall V. S. (1998). Embryonic stem cell lines derived from human blastocysts. *Science* 282 1145–1147. 10.1126/science.282.5391.1145 9804556

[B39] Tornero D. Tsupykov O. Granmo M. Rodriguez C. Gronning-Hansen M. Thelin J. (2017). Synaptic inputs from stroke-injured brain to grafted human stem cell-derived neurons activated by sensory stimuli. *Brain* 140 692–706. 28115364 10.1093/brain/aww347

[B40] Vogel S. Schafer C. Hess S. Folz-Donahue K. Nelles M. Minassian A. (2019). The in vivo timeline of differentiation of engrafted human neural progenitor cells. *Stem Cell Res.* 37:101429. 10.1016/j.scr.2019.101429 30933718

[B41] Wickersham I. R. Lyon D. C. Barnard R. J. Mori T. Finke S. Conzelmann K. K. (2007). Monosynaptic restriction of transsynaptic tracing from single, genetically targeted neurons. *Neuron* 53 639–647. 10.1016/j.neuron.2007.01.033 17329205 PMC2629495

[B42] Xiong M. Tao Y. Gao Q. Feng B. Yan W. Zhou Y. (2021). Human stem cell-derived neurons repair circuits and restore neural function. *Cell Stem Cell* 28 112.e–126.e. 32966778 10.1016/j.stem.2020.08.014 PMC7796915

[B43] Yamanaka S. (2007). Strategies and new developments in the generation of patient-specific pluripotent stem cells. *Cell Stem Cell* 1 39–49. 10.1016/j.stem.2007.05.012 18371333

[B44] Yu S. P. Tung J. K. Wei Z. Z. Chen D. Berglund K. Zhong W. (2019). optochemogenetic stimulation of transplanted iPS-NPCs enhances neuronal repair and functional recovery after ischemic stroke. *J. Neurosci.* 39 6571–6594. 10.1523/jneurosci.2010-18.2019 31263065 PMC6697405

